# Energetics and the immune system: Trade-offs associated with non-acute levels of CRP in adolescent Gambian girls

**DOI:** 10.1093/emph/eow034

**Published:** 2016-12-21

**Authors:** Heather Shattuck-Heidorn, Meredith W. Reiches, Andrew M. Prentice, Sophie E. Moore, Peter T. Ellison

**Affiliations:** 1 Human Evolutionary Biology Department, Harvard University, 11 Divinity Ave, Cambridge, MA 02139, USA; 2 Department of Anthropology, University of Mass Boston, 100 Morrissey Blvd, Boston, MA 02125, USA; 3 MRC Unit, The Gambia & MRC International Nutrition Group, London School of Hygiene & Tropical Medicine, Keppel Street, London WC1E 7HT, UK; 4 MRC Human Nutrition Research, Elsie Widdowson Laboratory, 120 Fulbourn Road, Cambridge CB1 9NL, UK

**Keywords:** life history theory, C-reactive protein, puberty, immune function, adipose tissue

## Abstract

**Background and objectives:** The human immune system is an ever-changing composition of innumerable cells and proteins, continually ready to respond to pathogens or insults. The cost of maintaining this state of immunological readiness is rarely considered. In this paper we aim to discern a cost to non-acute immune function by investigating how low levels of C-reactive protein (CRP) relate to other energetic demands and resources in adolescent Gambian girls.

**Methodology:** Data from a longitudinal study of 66 adolescent girls was used to test hypotheses around investment in immune function. Non-acute (under 2 mg/L) CRP was used as an index of immune function. Predictor variables include linear height velocity, adiposity, leptin, and measures of energy balance.

**Results:** Non-acute log CRP was positively associated with adiposity (β = 0.16, *P* < 0.001, *R*^2 ^= 0.17) and levels of the adipokine leptin (β = 1.17, *P* = 0.006, *R*^2 ^= 0.09). CRP was also negatively associated with increased investment in growth, as measured by height velocity (β = −0.58, *P *< 0.001, *R*^2 ^= 0.13) and lean mass deposition β = −0.42, *P *= 0.005, *R*^2 ^= 0.08). Relationships between adiposity and growth explained some, but not all, of this association. We do not find that CRP was related to energy balance.

**Conclusions and implications:** These data support a hypothesis that investment in non-acute immune function is facultative, and sensitive to energetic resources and demands. We also find support for an adaptive association between the immune system and adipose tissue.

## Teaser

How sensitive is the immune system to energetics? We find that Gambian adolescent girls who have high growth velocity invest less in non-acute immune function, and that there may be a non-pathological relationship between adipose tissue and innate immune function.

## BACKGROUND AND OBJECTIVES

The immune system is often considered in terms of its responses to pathogens, such as leukocyte proliferation, antibody production, or rapid increases in acute-phase proteins. These types of immune defenses are known to be energetically costly. Pyrogenic and non-febrile immune responses are associated with approximately 10% increases metabolic rate [1, 2], and acute immune responses are associated with reductions in activity, body mass and reproductive success in a wide range of animals [3]. However, acute responses do not represent the full metabolic cost of immune function. Normal immunocompetence is an active and dynamic process, in which large populations of cells and proteins undergo frequent turnover, with unknown associated energetic costs. In this paper, we use a life history perspective to investigate how energetic resources and demands are associated with variation in non-acute immune function.

Life history theory offers a way to understand the interactions between competing energetic demands, such as maintenance (including immune function), growth and reproduction [4, 5]. It is well-known that even slight changes in energy availability can influence investment in growth and reproduction [3, 6]. We also know that both acute and chronic immune responses can exhibit trade-offs with other life history demands. Acute inflammation is associated with decreased investment in linear growth in children [7, 8], and higher levels of inflammatory proteins predict reduced fecundity in women [9, 10]. Chronic illness is also associated with reduced growth and delayed puberty [11, 12]. Building on this body of research, we investigate how non-acute immune function relates to energy reserves, balance and investment in other life history demands.

In many respects, an acute immune response is a magnification of processes that are normally present in the body. For example, while CRP is often considered as part of the innate acute-phase response, it is also present in healthy individuals [13], where it circulates through the body and maintains immune surveillance for pathogens or other insults [14]. Upon recognition of pathogen or damage associated molecules, CRP initiates the complement system, promotes opsonization, activates leukocytes and stimulates the release of proinflammatory cytokines [15, 16]. As part of the innate acute response, CRP levels rise exponentially within 24–48 h after injury or infection. There is also variation in levels of non-acute CRP. Some of this variation is associated with obesity and has been linked to chronic disease [17]. It is also possible that a portion of the variation in non-acute CRP is related to energy availability and investment in competing life history demands.

We investigate the relationship between non-acute CRP and energetic factors such as growth and adiposity with data from adolescent girls in the Gambia. These pubertal girls are undergoing the adolescent transition and the accentuated energy demands of the growth spurt. During this time, individuals are increasing energy allocation to linear growth as well as differentially investing in tissue important to reproductive capacity, such as muscle and fat [18]. The immune system also undergoes changes during adolescence, though these are less well-understood. In terms of innate immune function, environmental factors that are known to increase inflammation in adults are also related to inflammation in adolescents, including social stress, sleep deprivation and obesity [19–21]. Additionally, the hormonal changes of adolescence have immunological consequences, with growth hormone and gonadal hormones exerting variable, and sometimes proinflammatory, effects on innate and adaptive immunity [22–24]. Despite the complexities of immune function in adolescence, this time period offers an intriguing possibility for exploring the energetic cost of non-acute immune function. Trade-offs between immune function and other demands may be especially evident in adolescents, as they are supporting increased growth rates as they finish maturation.

Working from our primary hypothesis that non-acute immune function is costly, we organize a series of analyses that test how CRP is related to energy reserves, energy balance and investment in growth. We predict that, even at very low levels of adipose tissue, girls who have more energy reserves will invest more in innate immune function. We also predict that being in positive energy balance, characterized by fat gain and by higher levels of C-peptide of insulin, will be associated with higher levels of CRP. Finally, we predict that girls who are investing more in growth will have lower levels of non-acute CRP. The ability of individuals to invest in growth, immune function or reproduction is a function of their individual energy status, and some girls may be able to support both increased investment in growth and high levels of investment in innate immune function [8]. Where appropriate, models will investigate how such phenotypic correlations may influence results, as well as whether individuals in different stages of the adolescent transition differ in investment strategy.

## METHODOLOGY

### Population

Study participants were 66 post-menarcheal adolescent females between 14 and 19 years at enrollment, residents of West Kiang, a rural province of The Gambia. This is an agricultural society with documented caloric and micronutrient constraints related to a highly seasonal environment [25]. Annually, there is a wet season (June–October), which coincides with the period of greatest agricultural labor, lowest food intake, and is characterized by population weight loss. There is also a dry season (November–May), in which relatively low-burden harvest work occurs, food intake is higher and weight is gained. Participants in the current study were born to mothers who were enrolled in a 1989–1994 protein, energy and micronutrient supplementation trial, and all participants were born during the wet season [26]. We contacted 81 adolescent females for participation in the study, and 12 refused (a 15% refusal rate). Of the remaining 69 women, three did not have serum available for CRP analysis and are not included in this report. At the time of the study, participants were not pregnant, had reported at least one menstrual cycle since parturition if lactating, and were not using hormonal contraception. Six participants had previously given birth and were breastfeeding at some point during the study. In mixed models accounting for repeat sampling across individuals, neither having given birth (β = 0.57, SE = 0.44, *P* = 0.19, *R*^2 ^= 0.01) nor currently nursing (β = 0.55, SE = 0.39, *P* = 0.16, *R*^2 ^= 0.01) were associated with log CRP.

**Figure 1.**
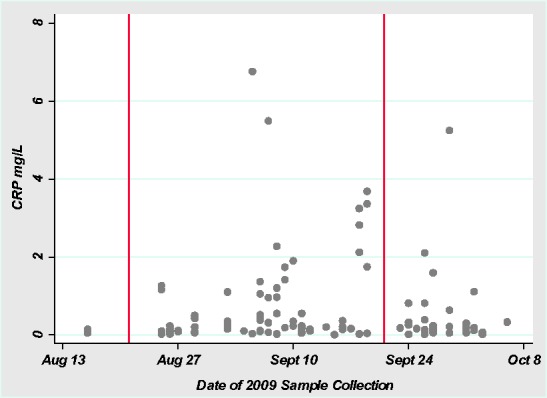
Ramadan and CRP in Season 1. CRP and sample collection date. Vertical lines indicate Ramadan period (Aug. 21–Sept. 21)

This study uses longitudinal data from three consecutive seasons, the fall 2009 wet season [July 30–October 3], the spring harvest season of 2010 [March 1–April 1] and the beginning of the fall 2010 wet season [July 14–August 12, 2010]. The majority of data collection in 2009 overlapped with Ramadan [August 21–September 21]. In this population, fasting associated with Ramadan is commonly observed after puberty, and is a daylight-hours fast lasting approximately a month. During Ramadan fasting, participants consumed their daily calories during their evening meal, after sunset. In this sample, the dates associated with Ramadan were associated with higher CRP levels (Fig. 1), and in a related study of the same subjects, both leptin levels and C-peptide of insulin levels were significantly higher during Ramadan than in the non-Ramadan wet season [27].

Within Season 1, samples collected strictly during Ramadan are not significantly different than samples collected outside of Ramadan (multilevel regression with individual as a random group effect and a Ramadan indicator variable predicting log CRP, β = 0.38, *P* = 0.20, *R*^2 ^= 0.02). However, if the Ramadan sampling period includes the week following Ramadan, there is a statistical difference (β = 0.94, *P* = 0.02, *R*^2 ^= 0.04). Our data suggest that Ramadan fasting may be associated with an increase in circulating CRP in non-obese adolescents with normal to high energy outputs, and that this effect may persist for several days following the end of Ramadan. However, there is not currently a substantive reason to include these days with our Ramadan period, and without them, Season is a superior explanation of the variation seen. Therefore, we include Season as a variable in our final models. Previous studies of the effect of Ramadan on CRP have variously reported that fasting lowers CRP in normal-weight individuals [28], that it raises CRP in normal-weight individuals but lowers CRP in obese persons [29], and that Ramadan fasting has no effect on CRP [30], and it is unclear whether our results are typical of Ramadan fasting in agricultural subsistence populations. The effects of fasting on circulating inflammatory proteins in normal-weight individuals, especially those living in rural and agricultural environments, deserves further study.

This data set includes longitudinal data across three seasons, with repeat samples for a majority of participants (Table 1). In total, we have 299 observations of CRP from 66 individuals. There are three seasons of data for 36 participants, two seasons of data for 16 participants, and one season for 14 participants. Most participants have two samples of CRP per season, although up to three participants in each season only have one sample available.

**Table 1.** Participant characteristics

	Season 1	Season 2	Season 3
	*N* = 56	*N* = 52	*N* = 45
Age (years)	17.2 (1.5)	17.4 (1.4)	17.5 (1.3)
BMI (kg/m^2^)	20.7 (2.8)	20.4 (2.8)	20.6 (3.1)
Adiposity (PCA metric)	0.07 (2.34)	-0.21 (2.40)	0.15 (2.34)
Height Velocity (cm/year)	0.70 (0.77)	0.79 (0.77)	0.89 (0.78)
CRP (mg/L)	0.66 (1.16)	0.33 (1.15)	0.18 (0.36)
Leptin (ng/mL)	14.74 (5.22)	13.56 (5.50)	11.25 (7.35)
C-peptide (ng/mg)	28.34 (22.82)	20.21 (15.42)	14.88 (10.34)
Time Since Menarche (years)	2.1 (1.6)	2.2 (1.3)	2.4 (1.3)
Weight (kg)	53.3 (8.0)	52.8 (7.9)	53.7 (7.9)
Height (cm)	160.44 (5.66)	160.87 (5.30)	161.33 (5.50)
Δ Weight in Season (kg)	−0.8 (1.4)	0.2 (1.2)	−0.3 (0.7)
Δ Lean Mass in Season (kg)	−0.5 (1.1)	0.3 (1.1)	0.1 (0.9)
Δ Fat Mass in Season (kg)	−0.3 (0.9)	0.0 (0.6)	−0.4 (0.6)
Skin Folds (mm)	14.5 (4.5)	18.2 (5.1)	19.1 (5.3)
Arm Fat (kg)	1.2 (0.6)	1.1 (0.7)	1.2 (0.7
Leg Fat (kg)	6.7 (2.5)	6.4 (2.6)	6.8 (2.6)
Trunk Fat (kg)	5.7 (2.6)	5.4 (2.6)	5.8 (2.5)
Android Fat (kg)	0.7 (0.4)	0.7 (0.4)	0.7 (0.4)
Gynoid Fat (kg)	3.2 (1.1)	3.0 (1.0)	3.2 (1.0)

Participant characteristics of 66 total adolescent Gambian girls across three seasons of data collection. All values are mean (standard deviation).

### Anthropometry and body composition

Height, weight and triceps skinfold thickness were measured in triplicate by the same trained observers. Height was measured for barefoot participants to the nearest millimeter with a stadiometer calibrated daily. Weight was measured in light clothing in triplicate to the nearest 0.1 kg on a battery-operated scale (Tanita Corporation, Japan), calibrated daily. Skinfold measures were taken with calipers, to the nearest 2 mm. Body composition was measured with the Tanita BC-418MA segmental body composition analyzer at the beginning and end of each sampling season. We used a population specific equation for the estimation of percent fat-free mass, validated to have a correlation with estimates from deuterium of R = 0.84 (95% CI 0.79–0.89) [31]. A modified version of this equation, which includes using triceps skinfold measures, was used to convert Tanita impedance readouts in Ohms into estimates of fat and lean mass. The Tanita measurements also provide information on the change of weight and body composition within season.

Whole and segmental body composition was measured at intake each season by DXA (Lunar DBX, GE Lunar Corporation, Madison WI). Limb regions are defined as the limbs of interest. Android and gynoid regions are based on definitions as in Lee et al. and Novotony et al. [32, 33]. Android regions are defined as the region from the top of the pelvis (bottom of the fourth lumbar) to a position equivalent of 20% of the distance between the pelvis and the femoral neck. Lateral boundaries are the lines that mark the arm regions. The gynoid region is a set distance (1.5 times the height of the android region) below the upper pelvis line. The lower boundary is twice the height of the android region below the upper boundary. The lateral boundaries are the lines that mark the leg regions.

Adipose tissue measures were tightly correlated, and we conducted a principal components analysis to create a global measure of adiposity. The variables included in this metric were BMI, trunk fat, arm fat, leg fat, gynoid fat and android fat. The eigenvalue of the first component was 5.39 times the size of the second, and accounts for 92.72% of the variance. No other eigenvalue was greater than 0.16. Accordingly, individual scores on the first principle component were used in subsequent analyses as measures of global adiposity.

**Figure 2.**
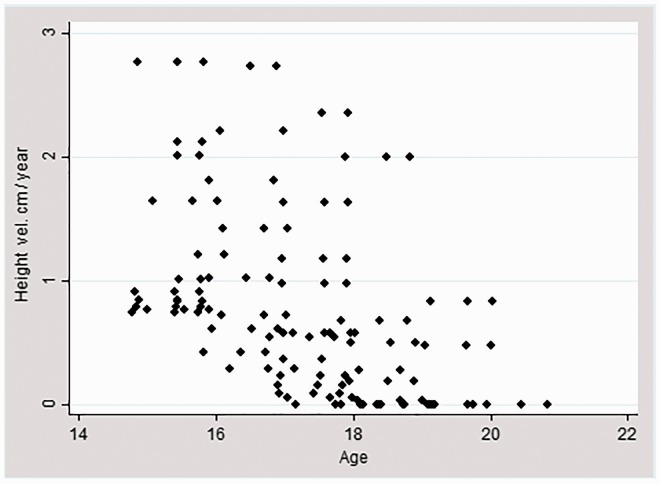
Linear height velocity by age. Height velocity decreases with increasing age. One outlier with a height velocity over 6 cm/year was excluded for this image

We calculated an average linear height velocity for each participant by averaging the change in height between each season, adjusting for duration of time between measurements. Two data points were identified as outliers and removed; one had an associated height velocity greater than 6 cm/year, more than twice the velocity of any other participant, the other exerted unusually high leverage due to high CRP and a high growth velocity. Nine individuals were present in multiple seasons and had a height velocity of 0 cm/year, indicating they were no longer growing. They were included the analyses. Previous studies of childhood and adolescent growth in the Gambia have demonstrated that, relative to British children, growth rates are lower at all ages, including a lower rate of peak growth velocity (averaging 6 cm/year in girls), and that both the age of peak height velocity (13.8 years) age of growth cessation are delayed [34]. Given this, and the rates of growth observed (Fig. 2), these data suggest some of these girls are still investing heavily in growth, while gradually coming to a point of growth cessation.

### Lab analyses

CRP and leptin were measured from serum derived from approximately 100 µl of peripheral whole blood collected by finger prick into microtainer capillary tubes. Samples were collected between 9:00 h and 11:00 h and were not fasted. CRP and leptin assays were run on site in the Gambia, and laboratory conditions were less stable, including daily temperature fluctuations, than in our normal lab at Harvard. Therefore, the inter-assay CVs are somewhat high. The assays for C-peptide of insulin were run in our Cambridge-based lab.

CRP was measured using a high-sensitivity ELISA protocol [35], which has a functional sensitivity of 0.00015 mg/L. Samples were run in duplicate, with an average intra-assay coefficient of variation of 5.2%. Samples that read as below the detection limit of 0.0001 mg/L, above 10 mg/L, or with coefficients of variation over 20% were rerun. The average intra-assay coefficient of variation for the low control was 2.4% and for the high control was 3.1%. The average inter-assay coefficient of variation for the low control was 23.7% and for the high control was 23.8% (*N* = 11). We explored the significance of these high CVs for our analysis through the inclusion of a dummy variable for plate. Results were unaffected. CRP values were not normally distributed, and were logged for analysis.

C-reactive protein is a part of the acute response, and it is necessary to exclude values indicative of acute inflammation. Unlike the chronically elevated CRP levels often seen in Western populations, here no individual had consistent CRP above 3 mg/L, though the CRP of one individual with a very high BMI for the population (26–28 over the seasons) did reliably fall around 1 mg/L. Given this, we use a conservative cut-off of 2 mg/L to exclude values indicative of acute inflammation, similar to other recent work [8, 10].

Leptin was analyzed with a commercially available 96-well plate leptin ELISA kit (BioVendor, Candler, NC), used according to kit protocol. The intra-assay coefficient of variation was 2.8% for the high control and 7.8% for the low control, and the inter-assay coefficient of variation was 16% for the high control and 50% for the low control (*N* = 10). While the CV for the low control was quite high, this represents only 2.3 ng/mL, and so is fairly minimal absolute variation. Leptin values were not normally distributed, and were logged for analysis.

C-peptide of insulin, a prohormone produced in a 1:1 ratio with insulin and secreted in urine, was measured in fasted first morning void urine samples dried on saturated filter paper by validated methods [36]. C-peptide of insulin was measured by radioimmunoassay according to kit protocols (Human C-peptide RIA kit manufactured by Millipore, catalogue number HCP-20K). Creatinine corrections were applied to adjust for urinary concentration [37]. The intra-assay coefficient of variation for the high control was 13.4% and for the low controls 13.8%. Inter-assay coefficients of variation were 10.1% for high and 15.7% for low controls (*N* = 13 assays). The sensitivity of the assay was 0.01 ng/ml. Analyses used C-peptide of insulin levels indexed to the individual, meaning normalized in reference to the average level for each individual.

### Statistics

Statistical analysis was performed in Stata 13. All models predict natural-log transformed serum CRP. As our longitudinal dataset contains repeat measures from the same individuals, the residuals are no longer independent. Models are linear mixed models, with individual as a random effect.

## RESULTS

### Associations between CRP and energy status

Over the study as a whole, participants were an average of 17.35 years old (sd 1.41), and 2.23 years post-menarcheal (sd 1.44). As continuous variables, in single predictor models, neither chronological age (β = −0.03, SE = 0.08, *P* = 0.75, *R*^2 ^= 0.01) nor time since menarche (β = 0.04, SE = 0.08, *P* = 0.59, *R*^2 ^= 0.03) were associated with CRP. While it is not significant, we include age as a covariate in our full models below.

**Figure 3.**
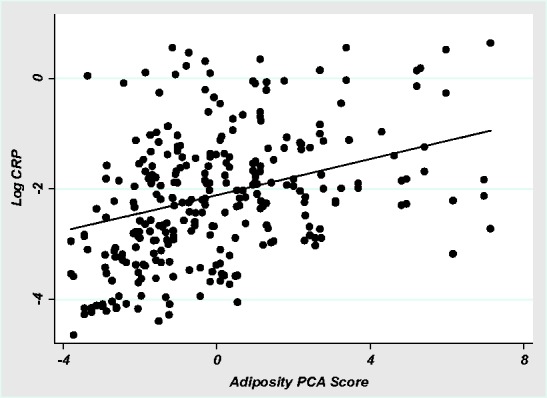
Log CRP by adiposity PCA score. Log CRP versus score on first component of adiposity PCA. Model is mixed-effects, with fixed-effects coefficient for adiposity component specifying the regression line (β = 0.16, *P* < 0.001, *R*^2 ^= 0.17)

The relationship between adiposity and CRP was present even in very lean individuals. In a single predictor, mixed model with individual random-effects, our global measure of adiposity positively predicted log CRP (β = 0.16, SE = 0.04, *P* < 0.001, *R*^2 ^= 0.17). Including individuals with CRP over 2 mg/L did not affect the model (β = 0.19, SE = 0.04, *P* < 0.001, *R*^2 ^= 0.17). Including age and season as covariates did not influence the significance of adiposity as a predictor of CRP (Model 1, Table 2). These results suggest a relatively continuous relationship between adiposity and CRP, even at very low levels of both (Fig. 3).

**Table 2.** Mixed model regressions predicting log CRP

Independent predictors	Coefficients and 95% confidence intervals					
	Model 1. Adiposity as predictor	Model 2. Leptin as an independent predictor	Model 3. Leptin as predictor	Model 4. C-peptide of insulin	Model 5. Linear growth velocity	Model 6. Full model with all predictors
Height velocity					−0.30 (−0.59, −0.2) *	−0.37 (−0.67, −0.07)*
C-peptide of insulin				0.31 (−0.03, 0.66) ns		0.33 (−0.01, 0.67) ns
Log leptin		1.17 (0.34, −1.99)*	−0.98 (−2.15, 0.18) ns			−1.32 (−2.49, −0.15)*
Adiposity	0.19 (0.11, 0.27)***		0.24 (0.14, 0.35)***	0.20 (0.11, 0.28)***	0.16 (0.08, 0.24)***	0.24 (0.13, 0.35)***
Season 2	−0.40 (−0.66, −0.13)**		−0.39 (−0.66, −0.13)**	−0.18 (−0.48, 0.12) ns	−0.36 (−0.65, −0.08)**	−0.20 (−0.51, 0.10) ns
Season 3	−0.69 (−0.97, −0.41)***		−0.86 (−1.20, −0.51)***	−0.42 (−0.78, 0.07)*	−0.58 (−0.89, −0.27)***	−0.59 (−0.98, −0.19)*
Age	0.02 (−0.11, 0.15) ns		0.00 (−0.12, 0.13) ns	0.02 (−0.13, 0.16) ns	−0.01 (−0.15, 0.15) ns	−0.04 (−0.19, 0.11) ns
Intercept	−2.11 (−4.34, 0.12) ns	−3.36 (−4.27, −2.45)***	−0.75 (−3.51, 1.99) ns	−2.62 (−5.16, 0.09)*	−1.58 (−4.21, 1.04) ns	0.11 (−3.17, 3.39) ns
*R* ^2^ model (overall)	0.24	0.09	0.25	0.23	0.26	0.28
Wald-χ^2^	44.96	7.69	47.72	36.31	48.86	52.23
*P*-value model	<0.001	0.006	<0.001	<0.001	<0.001	<0.001

Models are mixed model regressions, predicting log CRP, with individual as a random-effect.

* *P *< 0.05; **P < 0.001; ***P < 0.001.

The adipokine leptin also positively predicted CRP levels. Leptin values were logged for analysis, thus the coefficient is interpretable as indicating the percent change in CRP associated with a percent change in leptin. In a single predictor model, a 10% change in leptin levels was associated with an approximately 12% change in non-acute CRP (β = 1.17, SE = 0.42, *P* = 0.006, *R*^2 ^= 0.09, Model 2, Table 2). This association was strengthened if CRP values above 2 mg/L were included in the model (β = 1.69, SE = 0.48, *P* < 0.001, *R*^2 ^= 0.10). Our adiposity metric had a strong positive correlation with leptin, Pearson’s *R* = 0.69, *P* < 0.001. In models including adiposity, age and season as covariates, the association between leptin and CRP lost significance (Model 3, Table 2).

### Associations between CRP and energy balance

Contrary to our prediction that positive energy balance would be associated with higher investment in non-acute immune function, CRP did not differ significantly between individuals who were gaining fat within a season and those who were losing fat (β = 0.20, SE = 0.17, *P* = 0.24, *R*^2 ^= 0.01). This finding was not affected by including individuals with CRP above 2 mg/L in the model, or by including age, season and adiposity as covariates. Because prior work from this population found that older and younger individuals differ in how they prioritize tissue gain and loss, we considered whether pubertal transition stage may be influencing the relationship between energy balance and allocation to maintenance, and investigated models that included interaction terms between fat gain and age, as well as fat gain and a dichotomous growth velocity indicator (above or below 1 cm/year). We did not find any significant interactions between fat gain and these covariates of pubertal transition.

In a single predictor model, there was a positive relationship between CRP and C-peptide of insulin, a marker of insulin status and energy balance, though it did not explain much of the variation present (β = 0.44, SE = 0.15, *P* = 0.003, *R*^2 ^= 0.02). This relationship may be due to higher C-peptide of insulin and higher CRP in season 1, as when we included season as a covariate the relationship lost significance (Model 4, Table 2).

### Associations between CRP and investment in growth

**Figure 4.**
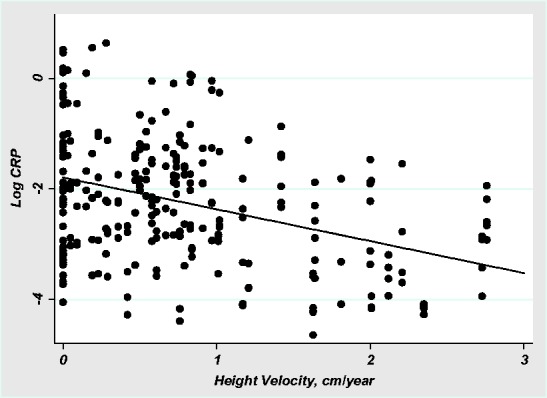
Log CRP by height velocity. Log CRP versus height velocity (cm/year). Model is mixed-effects, with fixed-effects coefficient for height velocity specifying the regression line (β = −-0.58, *P* < 0.001, *R*^2 ^= 0.13)

Adolescent girls who were investing more in growth had lower CRP values than individuals who were investing less in growth. The single predictor model indicates that height velocity was negatively associated with log CRP (β = −0.58, SE = 0.14, *P *< 0.001, *R*^2 ^= 0.13, Fig. 4). Including individuals with CRP results over 2 mg/L increased the strength of this association. In models including adiposity, age and season, height velocity continued to negatively predict CRP (Model 4, Table 2). These results were not affected by including individuals with CRP over 2 mg/L in the model. The association between current investment in linear growth and CRP did not extend to height. Neither height (β = −0.01, SE = 0.02, *P *= 0.75, *R*^2 ^= 0.0001), nor a height-for-age z-score (β = −0.03 SE = 0.16, *P *= 0.87, *R*^2 ^= 0.0003) were associated with CRP.

As an alternative metric of investment in growth, while in a single predictor model individuals who gained lean mass within a season had significantly lower CRP than those who were losing lean mass (β = −0.42, SE = 0.15, *P *= 0.005, *R*^2 ^= 0.08), this association lost significance (β = −0.25, SE = 0.15, *P *= 0.09) in models including adiposity, season, and age. Individuals who were gaining lean mass were also more likely to have higher growth velocity, though the correlation is relatively weak (Pearson’s *R* = 0.22, *P* = 0.001).

**Figure 5.**
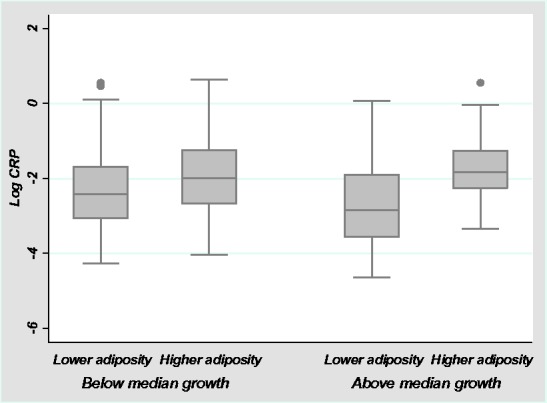
Relationships between log CRP, investment in growth and adiposity. Adiposity mediates the relationship between log CRP and growth. Girls who are investing heavily in growth and have low adipose reserves have significantly lower log CRP levels than the other groups in the figure

Adiposity appears to mediate the investment between immune function and growth (Fig. 5). Independent-group *t*-tests were conducted to compare mean log CRP levels between groups of girls based on investment in growth and adipose reserves, as defined by median splits for height velocity and global adiposity. Considering first girls who were investing less in growth, individuals with lower adiposity had lower CRP (M = −2.28, SD = 1.09) than individuals with higher adiposity (M = −1.87, SD = 1.09, *t* = 2.02, *P* = 0.05), though the difference becomes non-significant when including CRP values over 2 mg/L. Only eight girls were both investing heavily in growth and had high adiposity, however their CRP levels (M = −1.63, SD = 0.97) did not differ significantly from girls with similar levels of adiposity who were not investing in growth (M = −1.87, SD = 1.09, *t* = −1.10, *P* = 0.28). Girls who were investing in growth and had low adiposity had the lowest CRP levels of any group (M = −2.74, SD = 1.10), significantly lower than girls with low adiposity who were not growing (*t* = 2.19, *P* = 0.03) and girls who were growing but had higher adipose reserves (*t* = −5.09, *P* < 0.001).

In a full model including all predictors (height velocity, C-peptide of insulin, log leptin, adiposity, season and age), height velocity retains independent power for predicting log CRP (Model 5, Table 2).

## CONCLUSION AND IMPLICATIONS

As a lean population going through the pubertal transition under conditions of general energetic constraint, Gambian adolescent girls manifest the sorts of trade-offs in energetic investment that are predicted by life history theory. That theory stipulates that at each point in an organism’s life, energy is allocated between non-overlapping physiological categories in ways that optimize fitness [5, 38]. The adolescent transition is a particularly useful time to consider energetic dynamics, as during puberty investment in linear growth is first increased (the adolescent growth spurt) and then terminated. Meanwhile, investment in reproduction, manifested in females as the development of primary and secondary sexual characteristics and an increased metabolic priority given to energy storage as fat, increases as investment in growth subsides [18]. For instance, prior work from our group demonstrated that, in this participant sample, younger individuals prioritize lean mass, even at the expense of fat, while older individuals prioritize the fat deposits critical for reproductive success [39]. While the trade-off between growth and reproduction during the pubertal transition is readily observed and rationalized, attention is rarely directed at investment in maintenance over the same period. Our primary hypothesis here was that maintenance of the immune system might represent a substantial, flexible component of overall energetic costs, one that could be expected to covary with other energetic demands and resources during puberty.

In support of this hypothesis, we find evidence that increased allocation of energetic resources to growth may come at a cost to non-acute immune function, particularly in girls with low adipose tissue reserves. In this sample, girls who were growing faster and girls who were depositing lean mass had, on average, lower non-acute CRP. We interpret this as evidence that, in this energy-limited environment, these girls were subsidizing the increased costs of growth in early adolescence through decreasing investment in non-acute immune function.

We also find that, regardless of pubertal transition stage, individuals who had more available energy stores, as measured by a global adiposity metric and the hormone leptin, invested more in non-acute immune function. That adiposity was positively associated with CRP may seem unsurprising given the well-known relationships between obesity and inflammation [17]. However the inflammation induced by obesity is understood to result from pathological processes such as the increased recruitment of macrophages into adipose tissue in response to stress signals from enlarged adipocytes [40]. Here we find that CRP, in a range well below the chronic levels understood to result from obesity, is positively associated with adiposity, even in very lean subjects. We interpret this as evidence of a non-pathological relationship between adipose tissue and innate immunocompetence.

An adaptive relationship between adipose tissue and innate immunocompetence is congruent with several other lines of evidence. The fat body of insects regulates energy metabolism and storage and is also the site of immune and antibacterial responses [41, 42]. And it is well-known that a minimum level of body fat and the hormone leptin is necessary to maintain T-mediated immunity [43], and that leptin can regulate inflammatory immune processes, including stimulating Il-6 production in macrophages [44, 45]. This is congruent with our initial evidence that leptin positively predicts CRP, but here the relationship between leptin and CRP appears to be entirely mediated by adiposity. We also note the unexpected finding that in the final full model leptin negatively predicts CRP. In-vitro, leptin is reliable at stimulating inflammatory immune function (reviewed in [45]), but in human populations the relationship to immunity is often more complex. For instance, previous research in this population finds no relationship between leptin levels and acquired immune function in moderately undernourished children [46]. While the specifics of how adipose tissue influences CRP are complex, our results support a strong relationship between adiposity and innate immune function. An acute immune response imposes a substantial energetic cost in a relatively short time frame, and adipose reserves are critical for subsidizing immune responses in energy-limited environments. The innate immune system has been hypothesized to be more energetically costly than acquired immunity [47], and may be especially sensitive to energetic indicators. The relationships between adipose tissue, leptin and immune function suggest that organisms increase innate immune vigilance when adipose tissue reserves are sufficient to fund acute immune responses.

This framework, of considering the cost of an acute response as underlying the coupling of non-acute immune function to energetic reserves, also aids in interpreting why we do not find that energy balance, as measured by C-peptide of insulin or fat gain, was related to non-acute CRP. Positive energy balance does not necessarily correlate with large energy reserves. An organism could be gaining fat, or have a current caloric excess, but still have very low absolute levels of adipose tissue. While organisms may use markers of energetic balance to judge whether to embark upon long term energetic projects, such as female reproduction [6], an acute immune response will demand a large amount of energy in a short period of time [3]. Given this, it may be more reliable for organisms to use signals of energetic reserves to calibrate immune function, rather than energy balance.

Our findings suggest that there is a non-trivial metabolic cost either inherent in CRP production, or closely associated with levels of CRP. CRP has a half-life of 18 h [48], indicating that this population of proteins is undergoing frequent turnover, requiring some relatively constant commitment of energetic resources, though the metabolic cost of this is unknown. Our data also imply that there are meaningful differences in low levels of CRP. Interestingly, administration of human CRP to mice protects against pneumococcal infections, but not if CRP is administered after exposure to the bacteria, suggesting an early prophylactic function may be associated with CRP levels [49]. If CRP has a similar function in humans, our data would suggest that adolescence, in particular the period of the growth spurt, may be accompanied by increased risk of illness. While some pathogen infections are more frequent in adolescence [50], it is difficult to parse behavioral from physiological influences without direct measurement of the growth spurt. Alternatively, changes in levels of CRP may signify broader shifts in immunocompetence, perhaps particularly in innate immunocompetence, and not be specific to the action of CRP in particular. Outside of the context of chronic disease and pathology, the functional significance of variation in low-level CRP deserves study, as does the relationship between other aspects of immunocompetence, such as lymphocyte populations, and energetic demands.

In this work, we find that increased investment in growth is associated with reduced investment in non-acute immune function. Could investment in other energetic demands, such as reproduction or physical activity, also evidence trade-offs with non-acute immune function? Intriguingly, two recent studies support a hypothesis that there are some trade-offs between female reproductive function and higher levels of CRP. Though they do not specifically investigate non-acute CRP, these studies found that higher levels of CRP are associated with anovulation and lower levels of progesterone and estrogen [9, 10]. Concerning physical activity, vigorous exercise is known to suppress cell-mediated immunity and increase the risk of illness, while more moderate exercise generally has an anti-inflammatory effect [51]. How other types of physical activity, such as that associated with agricultural lifestyles, relates to immune function is an important locus for future research.

The dominant public health discourse concerning CRP is one of pathology and “chronic inflammation”, yet for at least a decade human biologists working in non-Western populations have found lower CRP than expected, and more complicated relationships between CRP and metabolic disease [52, 53]. We note that our CRP values are quite low in comparison to adult values from North America and Europe, though they are similar to those seen in other non-obese adolescent populations [54–58]. In our increasingly transnational world, understanding the significance of immunological biomarkers such as CRP, in both clinical and non-clinical settings, will require integrating knowledge of how different ecological contexts influence the manifestation of non-acute immune function.

While working with a subsistence agricultural population of pubertal girls has important benefits for understanding diversity in immune strategies, we should also keep in mind several limitations of this study. These include a relatively modest sample size and unequal representations of individuals across the seasons. We also cannot infer causality from these data. Our use of longitudinal data and a relatively stringent cut-off for acute CRP attempts to avoid biasing our data with individuals who are actively experiencing immune responses. However, it is possible that we are including some girls who have slightly elevated CRP and low growth because they have recently been sick. More broadly, it is unclear whether these relatively slight differences in CRP would be measurable in populations with higher rates of inflammation associated with obesity, or if these patterns would hold in populations living in environments with easy access to quick calories.

In summary, we contribute to a body of literature that emphasizes the importance of considering immune function as a metabolically expensive life history demand that may evidence sensitivity to energetic resources, and trade-offs with other demands. The constant production of cells and proteins required for immune vigilance is an important part of the energetic budget of organisms. The results of this paper support an understanding of investment in normal immunocompetence as a non-trivial energetic expense, one which may be drawn upon if needed to support other somatic strategies, and that may be tied to energetic reserves within the organism. We look forward to future research that aims to better quantify the cost of non-acute immune function.
